# Andreas Grimmel, Hrsg. 2020. Die neue Europäische Union. Zwischen Integration und Desintegration: Baden-Baden: Nomos Verlagsgesellschaft. 306 S., ISBN 978-3-8487-6428-0, 64,00 € (Print)

**DOI:** 10.1007/s12286-021-00475-8

**Published:** 2021-02-17

**Authors:** Florian T. Furtak

**Affiliations:** Hochschule für Wirtschaft und Recht Berlin, Alt-Friedrichsfelde 60, 10315 Berlin, Deutschland

In dem von *Andreas Grimmel* herausgegebenen, 12 Beiträge umfassenden Sammelband vermitteln namhafte Expertinnen und Experten der deutschen Europaforschung ihre neuesten sowohl empirisch gewonnenen als auch theoriegeleiteten Erkenntnisse zum europäischen Integrationsprozess. Der Sammelband richtet sich an einen europapolitisch interessierten Leserkreis in Forschung und Lehre sowie an Studierende an Universitäten und Hochschulen, die einen Master-Studiengang mit Schwerpunkt Europawissenschaften belegen. Nachfolgend wird exemplarisch auf einige der 12 Beiträge kommentierend eingegangen.

Der Titel „Neue Europäische Union“ mag auf den ersten Blick überraschen: Gibt es einen neuen EU-Vertrag, der den seit 2009 geltenden Vertrag von Lissabon ablöst, der Kompetenzen auf die EU-Ebene überträgt, die bislang den Mitgliedstaaten vorbehalten waren, der gar aus dem vom Bundesverfassungsgericht postulierten Staatenverbund einen föderalen Zusammenschluss formt, der den Idealen eines Ernesto Spinelli in seinem 1941 verfassten Manifest von Ventotene nahe oder zumindest näher kommt? Nein, ein neuer EU-Vertrag bildet für Grimmel und seine Mitstreiterinnen und Mitstreiter nicht die Grundlage für diesen Sammelband. Bei der Wahl des Titels geht es ihnen vielmehr darum aufzuzeigen, dass Integration und Desintegration keine sich ausschließenden Phänomene sind, sondern dass es vielmehr eine Gleichzeitigkeit von Integrations- und Desintegrationsdynamiken gibt, die eine neue Europäische Union (EU) begründen. In der Praxis zeigt sich das Gesicht dieser neuen EU darin, dass sich eine integrationsfördernde Politik und integrationshemmende Krisen parallel zu entwickeln vermögen. Und Krisen gibt es in der EU zu Hauf, worauf *Andreas Grimmel* in seinem Einführungsbeitrag hinweist: Brexit, Existenz EU-feindlicher Parteien im Europäischen Parlament, Uneinigkeit in der Migrationspolitik, finanz- und realwirtschaftliche Verwerfungen in der Eurozone und die Missachtung rechtsstaatlicher, demokratischer und liberaler Prinzipien durch Mitgliedstaaten. Nimmt man die seit Frühjahr 2020 herrschende Covid-19 Pandemie hinzu, lässt sich wahrlich von einer Poly-Krise sprechen, welche die EU vor eine große Herausforderung stellt.

*Christine Landfried* erläutert in ihrem Beitrag, wie sie sich die neue EU vorstellt: Eine bewusste Neugründung, die auf einem Konvent unter Beteiligung von Bürgerinnen und Bürgern beruht, um damit den Fehler beim 2003 einberufenen Konvent zur Erarbeitung eines europäischen Verfassungsvertrags nicht zu wiederholen, als fast nur Expertinnen und Experten beteiligt waren, was 2005 zu einer Ablehnung der Verfassung in Frankreich und den Niederlanden führte. Konkret fordert Landfried die Stärkung der kulturellen Grundlagen europäischen Regierens (wechselseitiges Einfühlungsvermögen, Solidarität, kooperatives Verhalten), eine Beendigung der nationalen Alleingänge in der Flüchtlingspolitik, Erhöhung der Eigeneinnahmen der EU durch Einnahmen aus einer Finanztransaktionssteuer und einer europäischen Unternehmenssteuer sowie eine stärkere Debatte über Ziele und Inhalte der EU-Politik in Öffentlichkeit und den Parlamenten. Landfrieds Forderung nach einer stärkeren Beteiligung der Öffentlichkeit ist bereits Programm der EU-Kommission. So startete bereits am 9. Mai 2020 die auf zwei Jahre angelegte Konferenz zur Zukunft Europas, deren Ziel es ist, die Meinung der Bürgerinnen und Bürger beim Handeln der EU besser zu berücksichtigen. Die Konferenz soll auf Bürgerdialogen aufbauen und die Debatten sollen sich an den politischen Prioritäten der EU wie Klimawandel und soziale Gerechtigkeit orientieren.

Der Beitrag von *Claudia Wiesner* knüpft an die Überlegungen Landfrieds an. Eine Demokratisierung der EU und die Ausbildung einer signifikanten input-Dimension sei durch eine Herausbildung bzw. Stärkung der europäischen Identität zu erreichen, was erforderlich mache, dass die demokratischen Institutionen und Verfahren der EU von einem europäischen Demos getragen werden. Diese Forderung wirft indes die Frage auf, ob die EU in Ermangelung eines durch eine kollektive Identität der Individuen konstituierten europäischen Demos überhaupt demokratiefähig ist.

Um Demokratie geht es auch *Andreas Grimmel* und *Ireneuz Pawel Karolewski* in ihrem Beitrag über „Democratic Backsliding in der EU“. Ihre These lautet, dass die EU Demokratie und Rechtsstaatlichkeit als Werte und nicht als rechtsverbindliche Regeln begreife, was sie zu einem schwachen Wächter von Demokratie und Rechtsstaatlichkeit mache. Und die drängendsten Herausforderungen für die EU lägen nicht mehr in der Legitimation europäischer Politik und der EU-Institutionen, sondern in den um sich greifenden Entdemokratisierungstendenzen einiger Mitgliedstaaten. Diese zeigen sich in der Tat in Polen und Ungarn, die beide bereits als etablierte Demokratien galten, wo sich jedoch in den vergangenen Jahren ein Trend zum Autoritarismus breitgemacht hat, indem die dort regierenden Parteien massiv die Axt an Gewaltenteilung, Rechtsstaatlichkeit und Pressefreiheit gelegt haben und weiter legen. Die Autoren bemängeln zu Recht, dass keine wirksamen Sicherungen gegen solche Tendenzen vorhanden seien, vor allem aber, dass es in der neuen EU eine deutliche Lücke zwischen dem politischen Anspruch und der rechtlichen Praxis dagegen vorzugehen gäbe. In diesem Zusammenhang verweisen sie auf Art. 7 EUV, der einen Stimmentzug eines Mitgliedstaats als Sanktion ermöglicht, sollte er die in Art. 2 EUV genannten Werte der EU schwerwiegend und anhaltend verletzen. Den beiden Autoren ist zuzustimmen, wenn sie kritisch anmerken, dass dieses Sanktionssystem völlig ungeeignet sei, um auf Verletzungen des Rechtsstaatlichkeitsprinzips in den Mitgliedsstaaten angemessen zu reagieren, weil es einer einstimmigen Entscheidung im Europäischen Rat bedarf. Kurzfristige Abhilfe ist hier jedoch nicht in Sicht: Die im November 2020 verabschiedete Verordnung des Rates und des Parlaments, die im Wesentlichen darauf abzielt, Mitgliedstaaten mit dem Entzug von Haushaltsmitteln zu sanktionieren, sofern sich Mängel in Hinblick auf das Rechtsstaatsprinzip negativ auf den Haushalt der EU auswirken, scheint zu kurz zu greifen, um die Einhaltung von Demokratie und Rechtsstaatlichkeit in der EU nachhaltig zu sichern.

Der Frage, inwieweit sich das Regieren im Mehrebenensystem verändert, um eine adäquate Antwort auf die Polykrise in der EU zu finden, widmen sich *Michèle Knodt, Martin Große Hüttemann* und *Alexander Kobusch*. Sie konstatieren am Beispiel der Wirtschafts- und Währungskrise sowie der Klimakrise, dass die „weichen“ Steuerungsformen zunehmend von einer „Härtung“ der Governance abgelöst werden, indem die Verbindlichkeit in den Politikfeldern durch verschiedene Maßnahmen verstärkt werde. Da Systemtransformationen jedoch nur graduell ausfallen, schlagen die Autoren vor, die Dichotomie zwischen Gemeinschaftsmethode als Form der harten Steuerung und Koordination als Form der weichen Steuerung zugunsten eines Kontinuums europäischen Regierens zu ersetzen, womit schrittweise Veränderungen besser zu erfassen wären. Von Interesse in diesem Zusammenhang wird sein, welcher Form der Governance die Bewältigung der Corona-Pandemie unterworfen sein wird, d. h., ob es auch hier ein Kontinuum zwischen intergouvernementaler und supranationaler Politikgestaltung geben wird. Blickt man auf die derzeitige Diskussion um die Beschaffung des Impfstoffs, scheint dieses Kontinuum zumindest gefährdet.

Mit der differenzierten Integration als ein zentrales Merkmal der neuen EU befasst sich der Beitrag von *Stefan Jagdhuber* und *Berthold Rittberger*. Sie argumentieren, dass die Methode Monnet bzw. die Gemeinschaftsmethode, die geprägt sei von einer schrittweisen für alle geltenden Ausweitung der Kompetenzen der Union, aufgeweicht werde durch das Prinzip der differenzierten Integration, welches eine politikfeldspezifisch, zeitlich und hinsichtlich der Mitgliedschaft flexible EU ermögliche. Begriffe in diesem Zusammenhang sind: Europa der zwei Geschwindigkeiten, Kerneuropa, Europa der variablen Geometrie, Europa der konzentrischen Kreise, Europa à la carte. Die Frage ist indes, ob das von den Autoren gezeichnete Modell der differenzierten Integration das Modell der einheitlichen Integration, das in die Präambel des EU-Vertrags durch das Ziel einer „ever closer union“ zum Ausdruck kommt, lediglich aufweicht oder bereits begonnen hat zu ersetzen? Denn die differenzierte Integration ist längst vertraglich verankert, und zwar durch die „Verstärkte Zusammenarbeit“ (Art. 20 EUV, Art. 326 AEUV), die es neun Mitgliedstaaten ermöglicht in einem Politikfeld einen stärkeren Integrationsgrad zu vereinbaren, was bereits in folgenden Bereichen praktiziert wird: Fiskalpakt, Scheidungsrecht, Finanztransaktionssteuer, Einheitspatent, Europäische Staatsanwaltschaft. Hinzu kommen die sog. opt-outs, die es Mitgliedstaaten qua EU-Vertrag ermöglicht haben nicht an allen Integrationsstufen teilzunehmen, wozu der Euro, Schengen, der Raum der Freiheit, Sicherheit und des Rechts, die Grundrechte-Charta sowie die Ständige strukturierte Zusammenarbeit (Art. 46 EUV) im Bereich der gemeinsamen Sicherheits- und Verteidigungspolitik zählen. Das Prinzip der differenzierten Integration biete, so die Experten, einen pragmatischen Ausweg, um Präferenzheterogenität und innenpolitischen Widerstand Rechnung zu tragen, indem es den Mitgliedstaaten selbst überlassen werde, in welchen Bereichen und in welchem Umfang sie sich an der EU-Politik beteiligen wollten. Dieser Befund ist zwar richtig, gleichwohl ist das Konzept der differenzierten Integration kein Allheilmittel, weil es zu viele Nebenwirkungen besitzt. So können zwar auf der einen Seite Mitgliedstaaten durch diese Flexibilität in der EU gehalten werden (bei Großbritannien hat das nicht geklappt, obwohl der ehemalige Premierminister David Cameron im Vorfeld des Referendums 2016 von der EU sogar eine Zusage über eine Abkehr vom Ziel einer „ever closer union“ erwirken konnte). Auf der anderen Seite jedoch kann aus der differenzierten Integration ein Flickenteppich von verschiedenen Integrationsstufen entstehen, der zur Unübersichtlichkeit führt und im Extremfall die Teilung der EU auf Dauer zementiert – mithin Desintegration die Folge ist.

Zur Rolle der EU in der Außen‑, Sicherheits- und Verteidigungspolitik schreibt *Gisela Müller-Brandeck-Bocquet*. Sie wirft vor dem Hintergrund der aktuell, insbesondere durch die Trump-Administration in den USA verursachten grassierenden Weltunordnung die Frage der Weltpolitikfähigkeit der EU auf. Anhand der vertraglichen Entwicklung führt sie aus, dass sich die EU Schritt für Schritt von der Gestalt eines außenpolitischen Zwerges gelöst, die Gestalt eines außenpolitischen Riesen indes noch lange nicht erreicht hat. Einen ähnlichen Befund konstatiert sie für die gemeinsame Sicherheits- und Verteidigungspolitik der EU. Doch es muss gerade im Interesse der EU liegen, so die Autorin, in diesen Bereichen mit einer Stimme zu sprechen, damit sie das Prinzip des Multilateralismus, auf das sie selbst angewiesen ist, unterstützen kann. Der Verwirklichung dieses Ziels dienen das Ausscheiden von Donald Trump als Präsident der USA und der Amtsantritt von Joe Biden, weil damit die Hoffnung verbunden ist, dass die regelentkernten internationalen Beziehungen, die u. a. durch die Kündigung internationaler Verträge durch die USA (Pariser-Klimaabkommen, Atom-Abkommen mit dem Iran) geprägt waren, der Vergangenheit angehören und durch eine neue Ära regelbasierter internationaler Beziehungen ersetzt werden. Und diese Chance muss auch die neue EU nutzen, indem sie, worauf Müller-Brandeck-Bocquet hinweist, nach Wegen zu häufigeren Mehrheitsentscheiden in der Gemeinsamen Außen- und Sicherheitspolitik sucht und ihr damit ein außenpolitischer Quantensprung gelingt.

Mit der Auswirkung von Krisen auf den europäischen Integrationsprozess befasst sich der Beitrag von *Darius Ribbe* und *Wolfang Wessels*. Sie konstatieren ein Kontinuum an Krisen während des gesamten Prozesses der europäischen Integration. Dabei sehen sie Krisen nicht als Gefährdung eines fortschreitenden differenzierten Integrationsprozesses, sondern als wesentliches Charakteristikum sowohl der alten als auch der neuen EU. Die These der beiden Autoren lautet, dass sich der Europäische Rat, also die Staats- und Regierungschefs der 27 Mitgliedstaaten zum Motor eines 4‑Stufen Modells einer vertikalen Integration entwickelt hat, welches durch die schrittweise Übertragung nationaler Kompetenzen, Souveränitäts- und Hoheitsrechte auf die europäische Ebene durch Änderung der Verträge gekennzeichnet sei. Anhand dreier Beispiele (Raum der Freiheit, Sicherheit und des Rechts, Gemeinsame Außen- und Sicherheitspolitik, Europäische Grundrechtecharta) zeigen sie auf, wie sich das 4‑Stufen Modell in der Vertragswirklichkeit ausgeprägt hat und welche Krisen zu den Integrationsschritten geführt haben. Im Ergebnis identifizieren sie Krisen als Triebfeder des Integrationsprozesses und die Staats- und Regierungschefs als Problemlöser und Antreiber einer schrittweisen Verlagerung der Problembearbeitung von der intergouvernementalen auf die supranationale Ebene. Der Einschätzung der beiden Autoren ist grundsätzlich zuzustimmen, gleichwohl sind insbesondere seit der Flüchtlingskrise 2015 verstärkt Tendenzen einer Renationalisierung zu beobachten.

Abschließend: Dem Herausgeber sowie den Autorinnen und Autoren ist ein informativer Sammelband gelungen, der die Forschung über Verlauf, Impulse und Hindernisse der europäischen Integration bereichert und vorantreibt, wobei jeder Beitrag für sich einen Erkenntnisgewinn bietet. Allerdings ist der Befund einer Parallelität von Integration und Desintegration im europäischen Integrationsprozess keine neue Erkenntnis. In diesem Zusammenhang sei auf den bereits 2013 von Annegret Eppler und Henrik Scheller herausgegebenen Sammelband „Zur Konzeptionalisierung europäischer Desintegration. Zug- und Gegenkräfte im europäischen Integrationsprozess“ sowie auf den in drei Sprachen 2019 von Jörn Axel Kämmerer, Markus Kotzur und Jacques Ziller editierten Band „Integration und Desintegration in Europa – Integration and Desintegration in Europe – Intégration et Désintégration en Europe“ verwiesen. Der Mehrwert des vorliegenden Bandes liegt indes in der systematischen und schlüssigen Erfassung der Gleichzeitigkeit von Integrations- und Desintegrationsdynamiken und den hierdurch gewonnenen Rückschlüssen für die Zukunft einer krisenbehafteten EU.

